# Intravital imaging of leukocyte behavior in atherosclerosis

**DOI:** 10.18632/aging.101792

**Published:** 2019-01-23

**Authors:** Wenjun Li, Jason M. Gauthier, Daniel Kreisel

**Affiliations:** 1 Department of Surgery, Washington University in St. Louis School of Medicine, St. Louis, MO 63110, USA; 2 Department of Pathology and Immunology, Washington University in St. Louis School of Medicine, St. Louis, MO 63110, USA

**Keywords:** atherosclerosis, experimental models, intravital two-photon microscopy, PET imaging, leukocytes

Atherosclerosis is a major cause of morbidity and mortality worldwide, clinically manifesting in peripheral vascular disease, cerebrovascular disease, or coronary artery disease. In the United States alone, coronary artery disease and stroke are responsible for around 370,000 and 140,000 annual deaths, respectively. Atherosclerosis is predominantly a disease of aging, as clinically significant plaques often develop over several decades. In fact, autopsy studies of children have demonstrated that the majority have early atherosclerotic lesions lining the aorta before 10 years of age. Despite the chronic nature of these plaques, it is being increasingly recognized that atherosclerosis is a dynamic process that may be reversible.

Over the last several decades our knowledge of atherosclerosis pathophysiology has expanded exponentially due to basic science investigations, most of which are heavily rooted in animal models of disease. In 2001, Reis and colleagues developed a model of atherosclerosis that has allowed for the study of plaque regression. In this model, plaque-bearing thoracic aortic segments from apoE-deficient mice that are fed a high fat diet and develop diffuse atherosclerosis secondary to hyperlipidemia, were interposed in the abdominal aorta of wild-type recipients. The aortic plaques significantly decreased in size after 9 weeks, which was accompanied by a reduction in “foam cell” macrophages [1]. The aortic heterotopic transplantation model was further perfected in 2003, when it was shown that transplantation of aortic arch segments that have aggressive lesions is a practical model for studying atherosclerosis [2]. The aortic arch transplant model was used by Llodra and colleagues to show that monocyte-derived cells traffic out of regressing plaques, whereas they are maintained in the subendothelium of progressing plaques [3]. Further investigations revealed that macrophages transport cholesterol out of these lesions via lymphatic vessels, reinforcing their critical role in plaque regression [4]. While these studies have greatly advanced our understanding how monocytic cells regulate atherosclerosis, they were limited by the inability to view these dynamic processes in vivo.

Intravital imaging has advanced our understanding of many immune-mediated inflammatory conditions. In 2011 intravital imaging was first applied to atherosclerotic arteries in order to visualize leukocyte recruitment in real time [5]. Later, Koltsova imaged aortic explants from apoE-deficient mice that were fed a high fat diet and observed interactions between CD4^+^ T lymphocytes and antigen presenting cells within lesions, which resulted in the secretion of pro-inflammatory cytokines [6]. Our laboratory has previously developed techniques to image leukocyte trafficking in beating heterotopic cardiac grafts with two-photon microscopy. In this model, a murine heart is transplanted into the neck of a recipient and the graft is stabilized, which enables intravital imaging for several hours. Appreciating the work done by Chevre and colleagues [7], where intravital two-photon imaging was applied to carotid artery atherosclerotic lesions, we developed a method of cervical aortic arch transplantation [8]. By transplanting plaque-bearing aortic arches from apoE-deficient mice into the neck of CX_3_CR1 GFP-reporter mice, we were able to stabilize the grafts and visualize the recruitment of CX_3_CR1^+^ monocytes into atherosclerotic plaques in real time, where they display only little motility and progressively decrease in number as the lesions regress. These findings are consistent with previous studies demonstrating emigration of monocyte-derived cells during atherosclerosis regression [3]. Using serial positron emission tomography (PET) imaging with a recently developed CCR2-targeted molecular probe, we found that the CCR2^+^ signal progressively decreases as plaques regress. Future intravital two-photon microscopy studies employing CCR2 reporter mice can delineate their recruitment, dynamic behavior and interactions with other cell populations within progressing and regressing plaques.

The impact of our findings is likely to be two-fold. By developing a model of atherosclerotic plaque regression that is amenable to intravital two-photon and PET imaging, we have developed a platform that will allow for the characterization of cellular pathways that regulate the evolution of plaques [1,3]. Visualization of dynamic leukocyte behavior and cellular interactions within atherosclerotic plaques of living mice was previously limited by technical hurdles. The transplant model further allows investigators to design experiments that distinguish between plaque-resident cell populations and cells that are recruited from the periphery. As such, our technique will surely empower future investigations aimed at elucidating the mechanisms of plaque progression and regression. Furthermore, our model allows for noninvasive serial imaging of atherosclerotic plaques with PET imaging. Our CCR2-targeted PET imaging may prove valuable to monitor responses to treatment over time. Thus, novel approaches to intravital imaging are warranted, as they may result in new diagnostic and therapeutic approaches for atherosclerosis that lessen the burden of this prevalent disease in the aging population.
